# First case of trans apical implantation of an aortic valve in a patient with dextrocardia

**DOI:** 10.1186/1749-8090-7-24

**Published:** 2012-03-13

**Authors:** Hellmuth S vH Weich, Jacques van Wyk, Wynand van Zyl, Rocco Vivier, Andre Phillips, Thomas Mabin

**Affiliations:** 1 Tygerberg Academic Hospital & Stellenbosch University, Cape Town, South Africa; 2 Panorama Mediclinic, Cape Town, South Africa; 3 Vergelegen Mediclinic, Cape Town, South Africa; 4 Division of Cardiology, Department of Internal Medicine, Private bag X1, Tygerberg 7505, South Africa

**Keywords:** Aortic valve, Replacement, Cardiac catheterization/intervention, Heart valve, Transapical, Percutaneous

## Abstract

We describe the clinical presentation and implantation procedure of the first transcatheter aortic valve implantation described in a patient with dextrocardia.

## Background

transcatheter aortic valve implantation is a relatively new technology where an aortic valve is implanted via a catheter [usually from the femoral artery]. It may also be implanted via a direct puncture of the left ventricular apex. We describe the first case of transcatheter aortic valve implantation [TAVI] in a patient with dextrocardia.

## Case presentation

The patient is a 66 year old female known with dextrocardia situs inversus. She also suffers from severe rheumatoid arthritis with multiple joint deformities and vertebral collapse due to steroid use. This has been treated with immunosuppressants over a prolonged period.

**Figure 1:**
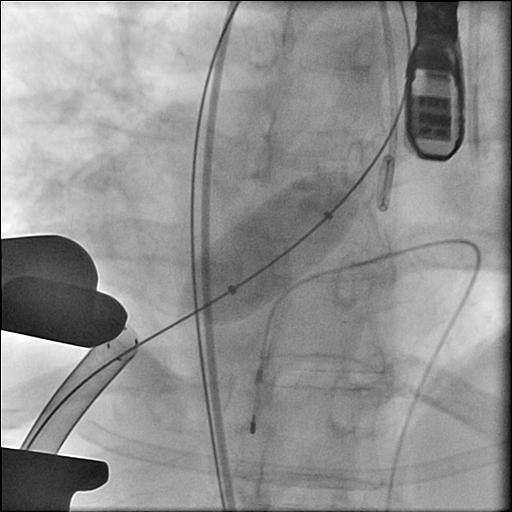
**Inflation of the balloon under rapid ventricular pacing**.

**Figure 2:**
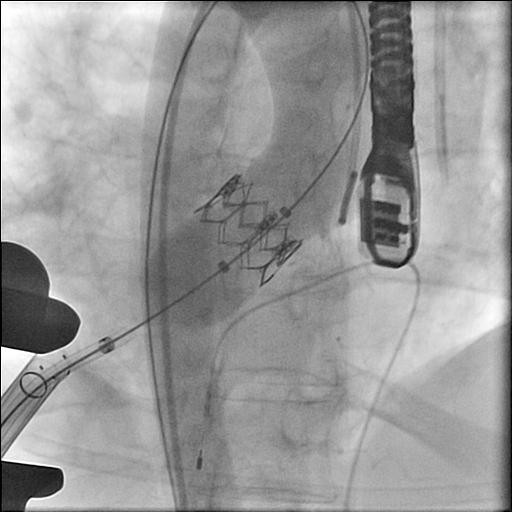
**Deployment of the valve**.

**Figure 3:**
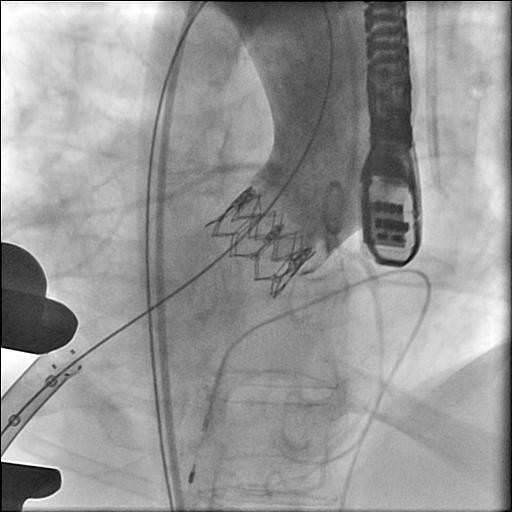
**Final result showing good position of the valve and patency of the coronaries**.

She presented to us with New York Heart Association class 3 dyspnoea. Echocardiography confirmed severe aortic stenosis with a mean gradient of 42 mmHg and a valve area of 0.8 cm^2^. We calculated her logistic EuroSCORE as 15% but the STS score was 14.5%. She was furthermore turned down for conventional surgery by a cardiothoracic surgeon due to frailty and was referred to our team for a TAVI. Peripheral vessels were diseased and a transapical approach was elected. Preparation of the theatre included switching around the positions of all the operators and theatre tables from our usual configuration: the first operator moved over to the right of the patient and the anaesthetist and anaesthetic machine, cardiologist, second surgeon and echo machine moved to the left of the patient. The case was done in a cath lab under general anaesthesia with constant trans esophageal echocardiography [TEE] monitoring. Trans thoracic echocardiography was used to localise the left ventricular apex and a mini-thoracotomy was performed. The apex was pre-closed with a purse string suture and the punctured with a needle. We encountered considerable difficulty crossing the diseased aortic valve and placing the stiff guidewire down into the descending aorta but ascribed this to the unfamiliar angles required to position it [see movie S1]. The valve was predilated without difficulty [see Figure 1]. A 23 mm Edwards SAPIEN XT™ valve on an Edwards Ascendra™ delivery system [Edwards Lifesciences™, Irvine, CA] was deployed under rapid ventricular pacing [see Figure 2][see movie S2]. Immediate evaluation with a supra-aortic contrast injection [see Figure 3] as well as TEE confirmed good valve function and minimal paravalvular incompetence. The delivery system was removed and the left ventricular apex closed with the purse string suture. The patient made an unremarkable recovery with discharge from hospital on day 4 post-operative. At her last follow-up 3 months post implantation, she was in NYHA class 1 and the prosthetic valve was functioning well.

## Conclusions

Dextrocardia is a rare condition and when not associated with Kartagener's syndrome, carries a normal life expectancy. It is therefore not unusual for patients to live up to ages where senile aortic stenosis is encountered. TAVI is a new technology requiring new skills for all operators involved and when this procedure is performed on a patient with dextrocardia, even more care should be taken to evaluate each step of the procedure for potential difficulties. We have however shown that with these precautions in place, this procedure is feasible.

## Consent

written informed consent was obtained from the patient prior to the procedure.

## Competing interests

The authors declare that they have no competing interests.

## Authors' contributions

Hw, TM, JvW, WvZ, RV & AP all contributed to the pre-operative planning of the case, the changes made to the layout of the theater and performed the procedure. HW acquired the images and wrote the initial manuscript. All authors read and approved the final manuscript

## Supplementary Material

Additional file 1 **Movie S1**. This movie shows some of the difficulty we experienced placing the wire down the descending aorta. Click here for file

Additional file 2 **Movie S2**. Deployment of the valve. Note that after contrast injection, the position is found to be too high and the device is pulled inferiorly prior to inflation of the balloon. This was done because the patient did not recover well from the first run of rapid pacing and we therefore preferred to reposition and deploy the valve in a single run of rapid pacing. Click here for file

